# System for Infectious Disease Information Sharing and Analysis: Design and Evaluation

**DOI:** 10.1109/TITB.2007.893286

**Published:** 2007-07-10

**Authors:** Paul Jen-Hwa Hu, Daniel Zeng, Hsinchun Chen, Catherine Larson, Wei Chang, Chunju Tseng, James Ma

**Affiliations:** 1 School of Accounting and Information SystemsUniversity of Utah Salt Lake City UT 84112-0090 USA; 2 Department of Management Information SystemsUniversity of Arizona Tucson AZ 85721-0108 USA; 3 Institute of AutomationChinese Academy of Sciences Beijing 100080 China; 4 University of Pittsburgh PittsburghPA 15260 USA

**Keywords:** Infectious disease informatics, outbreak detection, public health information systems, system evaluation

## Abstract

Motivated by the importance of infectious disease informatics (IDI) and the challenges to IDI system development and data sharing, we design and implement BioPortal, a Web-based IDI system that integrates cross-jurisdictional data to support information sharing, analysis, and visualization in public health. In this paper, we discuss general challenges in IDI, describe BioPortal's architecture and functionalities, and highlight encouraging evaluation results obtained from a controlled experiment that focused on analysis accuracy, task performance efficiency, user information satisfaction, system usability, usefulness, and ease of use.

## I. Introduction

Increasing globalization, combined with accelerating population mobility and more frequent travel, has made the prevention and management of infectious disease outbreaks a growing concern in public health. Emerging infectious disease and epidemic outbreaks are particularly important and represent critical challenges facing public health researchers and practitioners [1], [2]. In addition, potential threats of bioterrorism appear on the horizon [3].

Managing infectious disease outbreaks is intrinsically information intensive and requires substantial support for data gathering, integration, analysis, sharing, and visualization [4]. Such support requirements are becoming even more challenging because of the diverse, heterogeneous, and complex information available in enormous volumes and different sources that span jurisdictional constituencies both horizontally and vertically. Public health professionals such as epidemiologists can be better supported by advanced information systems (IS), as vividly manifested by emerging infectious disease informatics (IDI)—an interdisciplinary research area that focuses on the design, implementation, and evaluation of advanced systems, techniques, and methods for managing infectious disease and epidemic outbreaks, ranging from prevention to surveillance and detection [5], [6].

The design and implementation of an effective IDI system can be complex and challenging. At the data level, an expanding array of data that pertain to particular diseases, population characteristics, and related health considerations must be collected, organized, and archived, typically by different clinical institutions and health agencies. These data are heterogeneous in their semantics, modeling, granularity, aggregation, availability frequency, and coding/representation. Data sharing is critical to the pertinent institutions and agencies, which have to coordinate by explicitly specifying data ownership and access rights, as well as delineating the responsibilities associated with legal and privacy considerations. At the system level, these institutions and agencies often vary in their in-house systems, which adopt proprietary architecture designs and operate on different platforms. As Kay et al. [7] point out, most existing systems in public health have been developed in isolation.

The challenge and complexity of designing an IDI system extends beyond data and system heterogeneity. From the user's perspective, all relevant data must be seamlessly integrated to support his or her surveillance and analysis tasks that are critical to the prevention of and alert about particular disease events or devastating outbreaks. To be effective, an IDI system must encompass sophisticated algorithms for the automatic detection of emerging disease patterns and the identification of probable threats or events. An effective IDI system also must have advanced computational models that overlay health data for spatial–temporal analysis to support public health professionals' analysis tasks [8].

Several additional issues are crucial for system design and implementation, including the integration of multiple heterogeneous source data or systems, data accessibility and security, interfaces with geographic information systems (GIS), text document management support, and data or text mining. In particular, IDI design requirements include spatial–temporal data analysis and related visualization support. Typically, public health professionals approach the surveillance or detection of a probable outbreak as an event for which all related data are dotted and analyzed in spatial and temporal dimensions. Furthermore, the value of an IDI system is generally determined by the extent to which the system can present data and analysis results through intuitively comprehensible and cognitively efficient visualization. Ultimately, an IDI system must facilitate and enhance task performance by enabling public health professionals to use heuristics and preferred analysis methods to generate more accurate analysis results within a shorter time window.

**Fig. 1.**
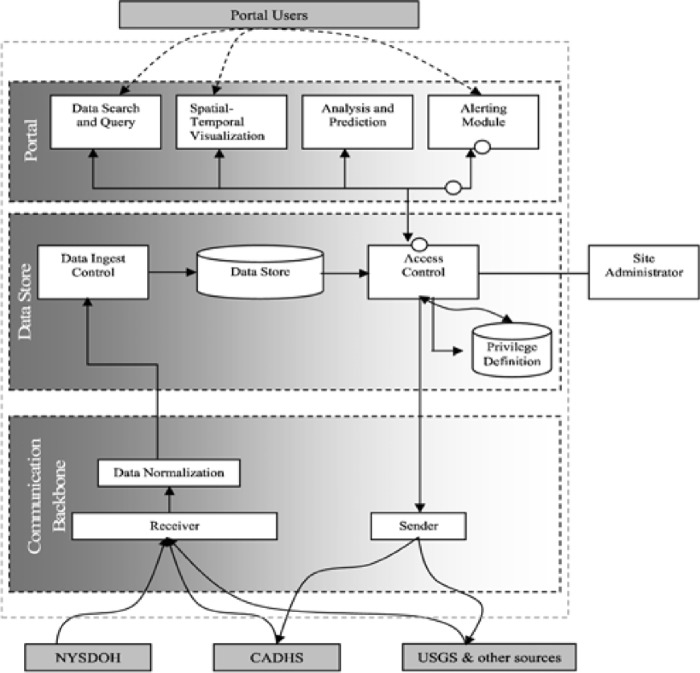
BioPortal system architecture.

To support the surveillance and detection of infectious disease outbreaks by public health professionals, we design and implement the BioPortal system, a web-based IDI system that provides convenient access to distributed, cross-jurisdictional health data pertaining to several major infectious diseases including West Nile virus (WNV), foot-and-mouth disease (FMD), and botulism. Our system development team is interdisciplinary, consisting of researchers in both IS and public health, practitioners, and officials from several state health departments. BioPortal supports sophisticated spatial–temporal data analysis methods, and has effective data/information visualization capabilities.

The rest of this paper is structured as follows. In Section II, we describe the architecture design of BioPortal and highlight its main technical components and functionalities. Next, in Section III, we discuss the value of BioPortal for infectious disease surveillance and management, derive hypotheses regarding its advantages and effects, and empirically test these hypotheses using a controlled experiment with 33 subjects. To assess BioPortal as a whole, we focus our evaluation on users rather than specific algorithms implemented as part of BioPortal and examine its effects on their task performances as well as subjective assessments of the system. In Section IV, we summarize our findings and note that our data support most of the hypotheses tested. Our results suggest that Bio Portal can better support public health professionals' analysis tasks, and is generally considered more usable, useful, and easier to use than the benchmark technology. Section V concludes with a summary, discussions of the paper's contributions and limitations, and some future research directions.

## II. BioPortal Architectural Design and Functionality

BioPortal is an integrated, cross-jurisdictional IDI infrastructure that has been running for testing and research purposes since early 2004 (see www.bioportal.org). Although it has not yet been adopted for operational use, it contains more than a dozen real-world data sets contributed by public health partners and other agencies. In this section, we summarize BioPortal's architectural design, its main components, data set availability, system functionality, and related outbreak detection research. The information that we present herein establishes the background for the evaluation study reported in the subsequent sections of this paper.

### A. BioPortal System Architecture

Fig. 1 illustrates the architecture of the BioPortal system, including the data flows between/among the main components of BioPortal as well as between BioPortal and external data sources. From a system's perspective, BioPortal is loosely coupled with state public health information systems in California and New York. It does not change the way these state systems operate. As needed, these systems transmit WNV/botulism information through secure links to the BioPortal using mutually agreed protocols. Such information is then stored in an internal data store maintained by the BioPortal. The system also automatically retrieves data items from sources, such as those from the USGS, and stores them in this internal data store.

All the system functions provided by BioPortal, including infectious disease data search and query, spatial–temporal visualization, outbreak detection analysis and related modeling, and automatic alert generation based on the results of outbreak detection analysis, are solely based on the data stored in the BioPortal internal data store, without further interactions with the contributing data sources. Technically speaking, we adopt a data warehousing approach, rather than alternative approaches such as query translation, information linkage, or terminology mapping [9] to address the distributed data integration challenges in IDI. This choice of approach is primarily based on the following characteristics of infectious disease data sources and associated analysis needs. First, unlike many other biomedical applications for which it has become increasingly easy to query data sources automatically from remote locations, most infectious disease data sets have been developed primarily for internal use. Although accessing the underlying databases through remote queries is technologically feasible, in practice, most IDI data providers are unwilling to “open up” their databases. Instead, they prefer pushing preprocessed data to (or preprocessing data waiting to be pulled by) a data warehousing system such as BioPortal while retaining full control over data fields to be shared (directly at the data level as opposed to at the data access control level). Second, the types of queries performed on IDI data sources typically are confined to data aggregation requests over particular geographical regions and time periods. Therefore, there is no need to strategize complex distributed queries. However, processing speed of the data aggregation is important because such operations must be carried out in large numbers for some outbreak detection analysis approaches (see Section II-C). Third, the amount of IDI data is relatively small in terms of storage volume because epidemiological information tends to contain a few short data fields, which makes a data warehousing approach feasible. Furthermore, overlaps between epidemiological data coverage are rare; therefore, the data warehousing effort becomes relatively manageable.

Internally, BioPortal consists of three main components: a web portal, a data store, and a communication backbone. In Section II-B, we provide the details of each component in more detail; here, we summarize BioPortal's implementation environment and the assumptions made on the user's end. BioPortal follows a standard three-tier web architecture. The data store component, developed using SQL Server, provides a data warehouse with information pulled from or pushed by contributing data sources. The communication backbone uses standard-compliant XML formats, and is built as multiple standalone Java applications that interface with various data sources using different messaging protocols. Most system functions are developed in Java using JSP pages to interact with the user. As such, all except one major component of BioPortal can be accessed by users through a standard Web browser. The exception is the visualization module, which is developed as a standalone Java application for improved performance, enhanced interactivity, and greater user interface control and is deployable through the Web Start technology (assuming that the Sun JRE environment is installed on the client machine).

### B. BioPortal System Components and System Functionality

Because the Web portal component of BioPortal implements the user interface and provides access to all main user functionalities—including: 1) searching and querying available infectious disease-related data sets; 2) visualizing the data sets using spatial–temporal visualization; 3) accessing analysis and outbreak detection functions; and 4) accessing the alerting mechanism—we do not discuss this component as one unit. Instead, we briefly summarize our work on 1) and 4) in this section, and then present the BioPortal visualization environment. Because data analysis and outbreak detection involve innovative spatial–temporal data mining research beyond system implementation, we defer their discussion to Section II-C.

#### 1. Portal Data Store

A main objective of BioPortal is to enable users from partnering states and organizations to share data. Typically, data from different organizations have different designs and are stored in different formats. To enable data interoperability, we use HL7 standards internally as the main storage format. Some data providers (e.g., New York state's HIN) have already adopted HL7 and can, thus, send HL7-compliant data to BioPortal directly. Additional steps are needed to ensure data interoperability for those data providers that do not yet have HL7-compliant data. First, we reach an agreement with them regarding the format (typically a simple home-grown XML format) for their data. Second, the data providers modify their data export module to implement this mutually agreed format. Third, when data from these providers reach BioPortal, a data normalization module maps the customized XML format on to HL7 using predetermined mapping rules implemented by the BioPortal team. In effect, the data from the HL7-compliant data providers also are processed by this module, because it removes from them unneeded data attributes, duplications, and common misspellings (based on a customized home-grown dictionary). This normalization module is not intended to resolve structural or semantic incompatibilities in an automated fashion; rather, it converts data to a predetermined format and performs shallow syntactic checking.

After being processed by the data normalization module, data are stored directly in BioPortal's main data store. This HL7 XML-based design provides a key advantage over an alternative design based on a consolidated database for which the portal data store must consolidate and maintain the data fields for all data sets. When an underlying data set changes its data structure, a portal data store based on the consolidated database must be redesigned and reloaded to reflect the changes, which severely limits system scalability and extensibility. To alleviate potential computational performance problems with our XML-based design, we identify a core set of data fields based on the queries that are likely to be performed frequently. These fields are extracted from all XML messages and stored in a separate database table to enable fast retrieval.

#### 2. Communication Backbone

The communication backbone component enables data exchanges between BioPortal and the underlying data sources. Several federal programs have been recently created to promote data sharing and system interoperability in the healthcare domain; the CDC's National Electronic Disease Surveillance System (NEDSS) initiative is particularly relevant for our research. It builds on a set of recognized national standards such as HL7 for its data format and messaging protocols, and provides basic modeling and ontological support for data models and vocabularies. The NEDSS and HL7 standards have had major impacts on the development of IDI systems. Although these standards have not yet been tested in cross-state sharing scenarios, they provide an appropriate foundation for data exchange standards in national and international contexts. BioPortal relies heavily on NEDSS/HL7 standards.

The communication backbone component uses a collection of source-specific “connectors” to communicate with contributing sources. We use the connector linking New York's HIN system and BioPortal to illustrate a typical design. The data from HIN to the portal system are transmitted in a “push” manner, i.e., HIN send through secure public health information network messaging system (PHIN MS) messages to the portal at prespecified time intervals. The connector on the portal side runs a data receiver daemon to listen for incoming messages. After a message is received, the connector checks for data integrity syntactically and normalizes the data. The connector then stores the verified message in the portal's internal data store through its data ingest control module. Other data sources (e.g., USGS) may have “pull”-type connectors that periodically download information from source Web sites, and examine and store those data in the portal's internal data store. In general, the communication backbone component provides data receiving and sending functionalities, source-specific data normalization, and data-encryption capabilities.

#### 3. Data Confidentiality and Access Control

Data confidentiality, security, and access control are among the key research and development issues for the BioPortal project. With regard to system development, programming and rules already developed for New York's HIN system constitute the main sources of our design and implementation decisions. Because there was no precedent for extending access to a data system across state lines, we needed to develop new access rules for BioPortal. We have created new security and user agreement forms for organizations with proposed access as well as individual users within those organizations. In addition, the agencies involved in developing BioPortal formally signed a memorandum of understanding prior to sharing any real data. The responsibilities of participating organizations and individuals with access include:

1) the establishment and definition of roles within the agency for access, and the determination of those individuals who fill those roles, including systems for termination of access;
2) security of data physically located on, or transported over the organization's network;
3) protection for the confidentiality of all data accessed, with prohibitions against disclosure of personal or health information to any other agency, person, or public media outlet; and
4) recognition of ownership rights of parties that have provided data.

The types of data that must be addressed separately with regard to access are data from humans or owned animals that require the highest levels of confidentiality, data from free-ranging wildlife, and data from other systems such as vectors (e.g., mosquitoes for WNV), land use, and so forth. The need for maximum access to track diseases must be balanced against the confidentiality concerns and risks of jeopardizing data reporting to the system. We summarize BioPortal's data coverage in Table I.

**TABLE I:** Infectious Disease Data Sets in BioPortal

Disease	Related Data Sets
West Nile virus	– Human (NY, CA '03); captive animal (NY '03); – Bird sightings (NY '01-'03, CA '03, USGS '99-'03); – Mosquito pool (NY '03, CA '00); mosquito treatment (CA '04) - Chicken sera (CA '03)
Botulism	– Adult (NY, CA '01-'02); infant botulism (national '04); – Avian botulism (USGS '99-'03)
Foot-and-mouth disease	– Middle Eastern countries (Iran '87-'03, Iraq '85-'02, Afghanistan '96-'03, Pakistan '85-'03, Turkey '85-'03); South America (Argentina '01)

#### 4. Data Search and Alerting

BioPortal provides limited data search functions to regular IDI users. Instead of developing a generic data search interface with a full range of search criteria, after extensive discussions with potential end users (i.e., state and county epidemiologists and public health researchers), we decided to concentrate on search criteria based primarily on location and time. A specialized tabular interface allows users to quickly identify infectious disease cases that occurred at certain locations within a specified period of time. Through this interface, the user can also get summary case counts across locations and times with different levels of granularity. An advanced search module is also available to power users. Using this module, a power user can build a personalized search interface that includes additional data-set-specific search criteria.

Because BioPortal can perform IDI data analysis automatically without user intervention, if potentially interesting events are detected, the concerned individuals (e.g., epidemiologists in charge) should be alerted. We are currently implementing three types of alerting mechanisms. The first one is by e-mail. The second is through the BioPortal Web interface, so when a user authorizes himself or herself on the BioPortal site and an alert message exists, a flashing icon will notify the user of the pending message. The third mechanism is cellular phone notification through an automated Web-based short text message interface for urgent alerts.

#### 5. Data Visualization

An important role of visualization in the context of large and complex data set exploration is to organize and characterize the data visually to assist users in overcoming information overload problems [10]. BioPortal makes available an advanced visualization module, called the spatial temporal visualizer (STV), to facilitate the exploration of infectious disease case data and summarize query results. Developed as a generic visualization environment, STV can be used to visualize various spatial–temporal data sets simultaneously.

**Fig. 2.**
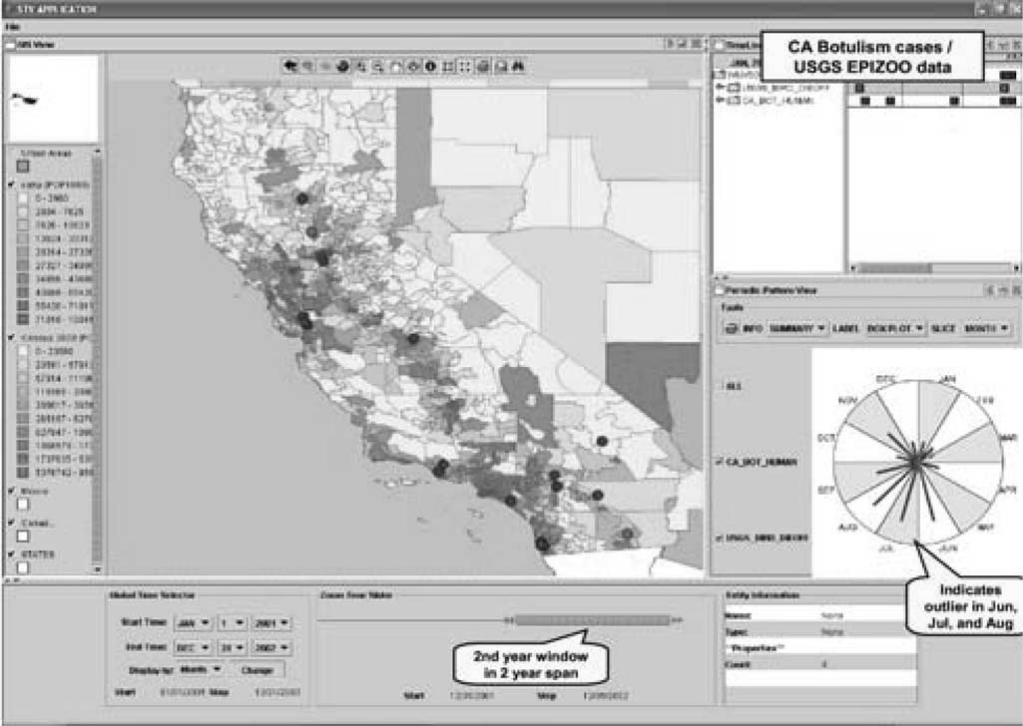
Spatial–temporal visualization in bioportal.

The STV has three integrated and synchronized views: periodic, timeline, and GIS. The periodic view provides the user with an intuitive display to identify periodic temporal patterns. The timeline view provides a two-dimensional timeline, along with a hierarchical display of the data elements organized as a tree. The GIS view displays cases and sightings on a map. Fig. 2 illustrates how these three views can be used to explore infectious disease data sets; the top left panel shows the GIS view. The user can select multiple data sets to be shown on the map in a layered manner using the checkboxes (e.g., disease cases, natural land features, land-use elements). The top-right panel corresponds to the timeline view and displays the occurrences of various cases using a Gantt chart-like display. The user can also access case details easily using the tree display located to the left of the timeline display. Below the timeline view is the periodic view with which the user can identify periodic temporal patterns (e.g., months with an unusually high number of cases). The bottom portion of the interface allows the user to specify subsets of data to be displayed and analyzed.

As discussed in Section II-A, to achieve fine-graded interface control and high interactivity, STV has been developed as a standalone Java application, which can be deployed transparently across the Web. The essential data elements (location, time, and event type) displayed by STV are all captured in the relational tables in the BioPortal internal data store. The auxiliary data elements (e.g., case details, needed only when a user wants to learn more about a particular data point) may be retrieved from the HL7 XML messages stored in the BioPortal internal data store. Because STV is executed on the client machine, real-time data transmissions between the client machine and BioPortal server are necessary. For better performance and shorter response time, STV caches much of the data needed on the client side.

### C. Outbreak Detection and Spatial–Temporal Data Analysis

In addition to data access, query, and visualization, BioPortal provides data analysis capabilities, particularly in the area of spatial–temporal data analysis. In IDI applications, measurements of interest such as disease cases are often made at various locations in both space and time. In recent years, interest has increased in answering several central questions, which have great practical importance in outbreak detection and arise from spatial–temporal data analysis and related predictive modeling: How can areas with exceptionally high or low measures be identified? How can observers determine whether unusual measures can be attributed to known random variations or are statistically significant? In the latter case, how should the explanatory factors be assessed? How can statistically significant changes be identified in a timely manner in specific geographic areas? For instance, unusual clustering of dead birds has been proven to be highly indicative of WNV outbreaks.

From a modeling and computational perspective, two distinct types of spatial–temporal clustering or hotspot analysis techniques have been developed. The first type is based on various kinds of scan statistics, and has been used with increasing frequency in public health and infectious disease studies [11]. The second type is based on data clustering and its variations, and has found successful application in crime analysis [12]. BioPortal makes both types of methods available through its Web interface. In addition, it allows the user to interactively invoke these methods and visually inspect their results through STV.

One major computational problem faced by existing methods is that the shapes of potential hotspots are limited to simple, fixed symmetrical shapes for analytical and search efficiency reasons. As a result, when the real underlying clusters do not conform to such shapes, the identified regions are often poorly localized. To overcome this major computational limitation, as part of the BioPortal technical research effort, we have developed an alternative and complementary modeling approach called risk-adjusted support vector clustering (RSVC). Hotspot analysis differs from standard clustering in that clustering must be performed relative to baseline data points (representing a “normal” situation). In RSVC, we apply the “risk adjustment” concept from a crime hotspot analysis approach [12] to incorporate baseline information in the clustering process. The basic intuition behind RSVC is as follows: A robust, SVM-based clustering mechanism called support vector clustering allows detection of clusters with arbitrary shapes based on the distances defined over pairs of data points. By adjusting distance measures proportionally to the estimated density of the baseline factor, areas with high baseline density make it more difficult to group data points together as clusters, because the distances between these data points have been adjusted upward. We have also extended our RSVC approach to perform prospective hotspot analysis aimed at monitoring data sources on a continuous basis. For technical details of this BioPortal spatial–temporal data analysis work, interested readers are referred to [13] and [14].

## III. Hypotheses and Evaluation Design

We conducted a controlled experiment to evaluate BioPortal holistically. Our foci were objective user task performance and subjective self-reported system assessments. Our evaluation did not involve algorithmic assessments or examine individual components of BioPortal (e.g., hotspot analysis and outbreak detection), which have been studied previously [14]. In this section, we discuss the hypotheses tested and detail our evaluation design.

### A. Hypotheses

We followed the system success evaluation framework by DeLone and McLean [15], and focused on evaluating the essential system characteristics of BioPortal and its impacts on user task performance measured objectively by analysis accuracy and task completion efficiency. We also examined users' self-reported assessments of BioPortal in terms of satisfaction, usability, usefulness, and ease of use, all of which are critical in system evaluations [16]. For benchmark purposes, we included a computer-based spreadsheet program commonly used by public health professionals in their analysis tasks.

#### 1. Analysis Accuracy

By integrating interrelated data extracted from different sources and presenting them in a visually intuitive and comprehensible way, BioPortal can be expected to better support various analysis tasks by public health professionals. Therefore, we tested the following hypotheses. $H1A:$ The analysis accuracy that results from the use of BioPortal is higher than that associated with the benchmark spreadsheet program. $H1B:$ The accuracy improvement that results from the use of BioPortal, as compared with the benchmark spreadsheet program, increases with task complexity.

#### 2. Task Completion Efficiency

By providing convenient access to integrated data extracted from difference sources, together with easy-to-use analytical algorithms and effective visualization, BioPortal can be expected to make public health professionals increasingly efficient in their task performance. We, therefore, tested the following hypothesis. $H2:$ The task completion efficiency associated with BioPortal is higher than that observed with the benchmark spreadsheet program.

#### 3. User Satisfaction

User satisfaction is a fundamental aspect of system evaluation and embraces user information satisfaction that emphasizes information requirements [17]. Because of the critical importance of information support in an IDI system, we explicitly focused on user information satisfaction and tested the following hypothesis. $H3:$ The user information satisfaction that results from the use of BioPortal is significantly higher than that observed with the benchmark spreadsheet program.

#### 4. System Usability

System usability has been shown to affect user adoption, system usage, and satisfaction [18]. Several usability instruments have been developed and validated [19], [20]. Of particular importance is the user interaction satisfaction (QUIS) scale [19] capable of assessing a system in five fundamental usability dimensions—overall reactions to the system, screen layout and sequence, terminology and system information, system learnability, and system capabilities. We tested the following hypothesis. $H4:$ BioPortal is more usable than the benchmark spreadsheet program and shows favorable usability scores in overall reaction to the system, screen layout and sequence, terminology and system information, system learnability, and system capabilities.

#### 5. Perceived Usefulness

System usefulness is critical to voluntary use of a new system [21], [22], and generally refers to the extent to which an individual considers a system useful in his or her work role. BioPortal offers effective data integration support, and has sophisticated built-in functionalities and intuitive visualization designs; as a result, it can be expected to better support the demanding information processing often required in an analysis task. Hence, we tested the following hypothesis. $H5:$ The usefulness of BioPortal, as perceived by an individual, is significantly greater than that of the benchmark spreadsheet program.

#### 6. Perceived Ease of Use

Perceived ease of use refers to the degree to which an individual considers his or her use of a system to be free of effort [21]. Ease of use represents an essential motivation for individuals' voluntary use of a system [23], and can affect their adoption decisions significantly [22]. Hence, we tested the following hypothesis. $H6:$ The ease of use of BioPortal, as perceived by an individual, is significantly greater than that of the benchmark spreadsheet program.

### B. Evaluation Design

We adopted a randomized, between-groups design. Our subjects were graduate students attending the management school or the public health school of a major university located in the southwestern United States. All subjects were knowledgeable about computer-based spreadsheets but varied substantially in general public health knowledge. Each subject was randomly assigned to use one particular system (BioPortal or the spreadsheet program), though we remained mindful of maintaining a balance in the subject-technology assignment.

With the assistance of several experienced public health researchers and professionals, we created six analysis scenarios common in public health and then developed a total of 11 experiment tasks accordingly. The assisting experts classified the experiment tasks on the basis of complexity: low, medium, or high. A complete listing of the scenarios and analysis tasks used in the experiment is available upon request. We provide two examples as follows.

*Scenario 1:* Examine data related to WNV.

*Task 1:* In 2002, which county in New York had the highest dead bird count? (complexity $=$ low)

*Task 2:* Of the three listed bird species, Bluejay, Crow, and House Sparrow, which had the highest number of positive cases of WNV? (complexity $=$ low)

*Scenario 6:* Determine correlations between the incidence of WNV and dead bird occurrences and mosquito pool counts.

*Task 10:* Using the BioPortal system or the spreadsheets, as assigned, to investigate WNV disease, can you determine whether, during 2002, there is a correlation between the dead bird occurrences and mosquito pool counts? (complexity $=$ high)

*Task 11:* (Continued with Task 10) If so, what correlation do you observe? (complexity $=$ high) To assess an individual subject's accuracy in each task, we consolidated the analyses by the assisting experts to establish a “gold-standard” solution for that task. We measured analysis accuracy using a ten-point scale, with one being completely incorrect and ten being completely correct. We measured task completion efficiency by using the amount of time that a subject needed to complete a task. We evaluated user information satisfaction [17] using a seven-point Likert scale, with one indicating extreme disagreement and seven indicating extreme agreement. We adapted question items from previous research [21] to measure system usefulness and ease of use, using a seven-point Likert scale with one indicating extreme disagreement and seven indicating extreme agreement. We adopted the QUIS instrument [19] with a nine-point Likert scale to evaluate system usability. ^1^^1^Details of the scale used in QUIS are available in [19]. In general, lower scores represent more favorable usability assessments (e.g., easy, wonderful, clear) than higher scores (e.g., difficult, terrible, confusing), with one being most favorable and nine being the most unfavorable.

Before the experiment, we used a script to inform the subjects explicitly of our objective and data analysis plan while ensuring them of the necessary information privacy. Subjects were asked to provide some demographic information, and self-assessments of their general computer self-efficacy and knowledge about computer-based spreadsheets and public health. We provided each subject with an overview of his or her assigned system and a training session based on sample tasks to illustrate how to use that system. In the experiment, each subject was asked to complete all analysis tasks grouped by analysis scenario and sequenced in increasing complexity, i.e., tasks progressing from low to high complexity. After completing all the tasks, each subject had to complete a questionnaire survey to provide his or her assessment of the system's usability, usefulness, and ease of use, as well as his or her satisfaction with the information support by the system. We imposed a 50-min time limit in the experiment, which was appropriate according to the results of a pilot study [24].

## IV. Results and Discussion

A total of 33 subjects voluntarily participated in the experiment. Among them, 17 subjects used BioPortal, and the remainder used the spreadsheet program. Of those using BioPortal, 9 subjects had high domain knowledge and the others were low in domain knowledge. A similar distribution was observed in the spreadsheet group. According to our analysis, the subjects in the BioPortal and spreadsheet groups are comparable demographically, and reported similar self-assessments in general computer efficacy and computer-based spreadsheets.

We reexamined the reliability of our instrument by assessing its internal consistency [25]. As summarized in Table II, the subjects' evaluative responses showed that almost all constructs exhibited a Cronbach's alpha value exceeding the commonly suggested threshold of 0.8 [26], thus, suggesting adequate reliability of our instrument.

**TABLE II:**
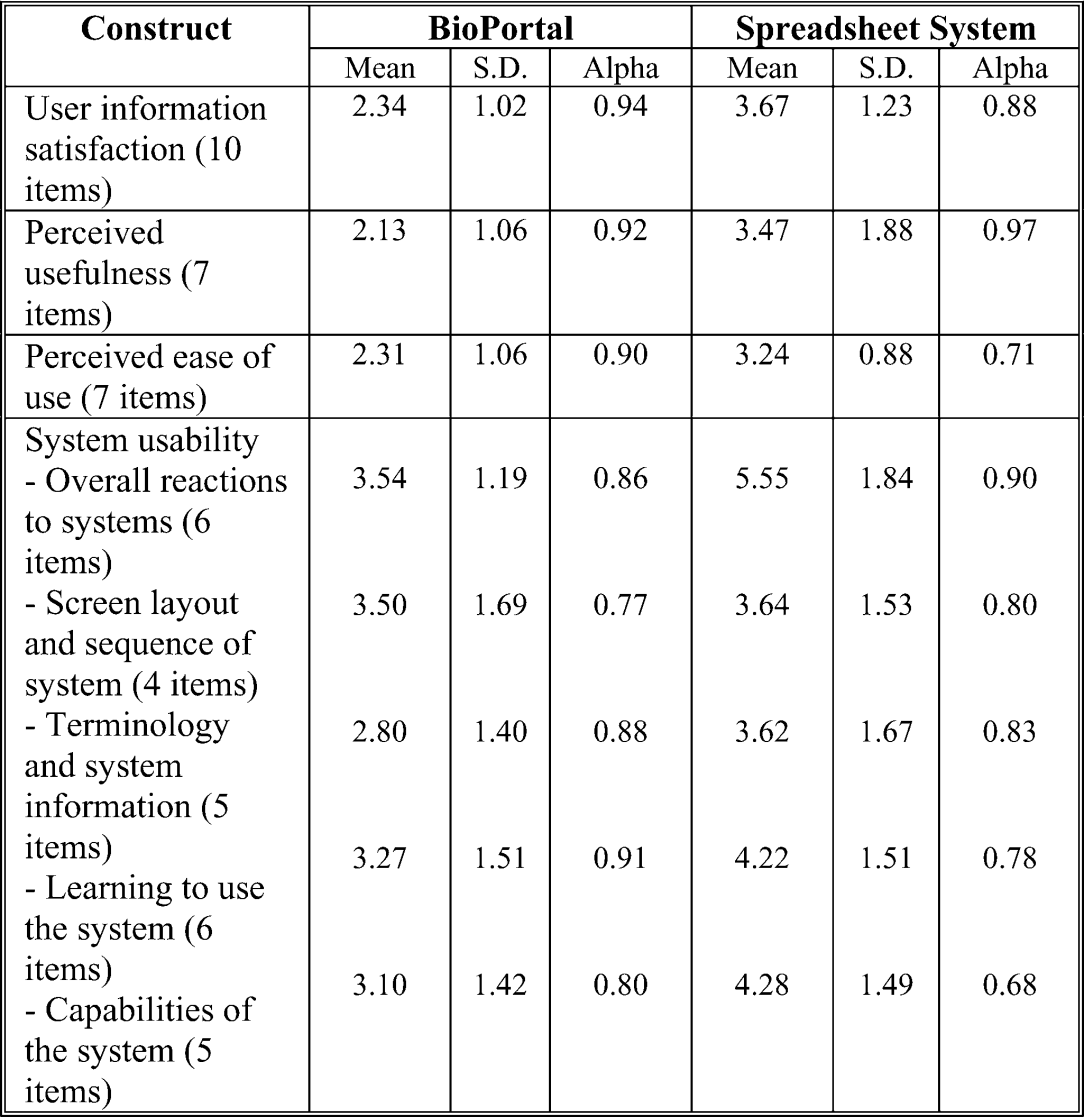
Summary of Descriptive Statistics and Construct Reliability Analysis

We tested the main effect of system (BioPortal *versus* the spreadsheet program) and domain knowledge (low *versus* high general public health knowledge) as well as their combined effects by performing an analysis of variance (ANOVA) with each dependent variable on the basis of subjects' responses. We also performed a paired t-test to assess the difference in each dependent variable obtained from the subjects using BioPortal *versus* the spreadsheet program.

### A. Effects on Analysis Accuracy

We used the gold-standard result to evaluate the accuracy of each task performed by subjects. For each subject, we aggregated his or her analysis accuracy across all the tasks performed in the experiment and used the overall accuracy to test the hypothesized main effect of system. According to our analysis, the system had a significant effect on analysis accuracy ($p$-value $< 0.01$). We further investigated the effect of system on the basis of task complexity, and found that the system's effect on analysis accuracy was insignificant for low-complexity tasks but significant for tasks of medium and high complexity. BioPortal's accuracy was greater $(\hbox{mean} = 81.94, \hbox{SD} = 21.23)$ than that of the spreadsheet program $(\hbox{mean} = 61.19, \hbox{SD} = 17.92)$, and the difference was significant at the 0.01 level. Thus, our data supported H1A and H1B.

### B. Effects on Task Completion Efficiency

Based on our analysis, the *system showed* a significant main effect on task completion efficiency ($p$-value ${<}0.01$). We compared the amount of time required to complete a task using the respective systems and found that, on average, subjects using BioPortal could complete an analysis task considerably faster (mean $= 36.28$ min, SD $= 11.33$ min) than their counterparts supported by the spreadsheet program (mean $= 48.23$ min, SD $= 5.07$ min); the difference was significant at the 0.01 level. Thus, our data supported H2.

### C. Effects on User Information Satisfaction

According to our analysis, the main effect of the system on user information satisfaction was significant statistically ($p$-value ${<}0.01$). Overall, subjects using BioPortal exhibited higher satisfaction with the information support (mean $= 2.34$, SD $= 1.02$) than their counterparts supported by the spreadsheet program (mean $= 3.68$, SD $= 1.23$); the difference was significant at the 0.01 level. Thus, our data supported H3.

### D. Comparative Analysis of System Usability

According to our analysis, the system had a significant main effect on both overall reactions to the system ($p$-value ${<}0.01$) and system capabilities ($p$-value ${<}0.05$) but not on screen layout and sequence or terminology and system information. The effect on system learnability was somewhat significant statistically. Overall, our subjects considered BioPortal generally usable and recognized its utilities for supporting their analysis tasks. Our evaluation results indicated that the design of BioPortal may need to improve in screen layout and sequence, as well as in language (e.g., clarity and user friendliness). Our subjects considered their learning to use BioPortal not particularly difficult, but its learnability could be enhanced further. According to our comparative analysis of subjects' self-reported assessments of the respective systems, BioPortal arguably was more usable than the spreadsheet program in most, but not all, fundamental usability dimensions, though the between-groups differences are not statistically significant. Thus, our data partially supported H4.

### E. Effects on Perceived Usefulness

Our analysis shows that the system had a significant effect on perceived usefulness ($p$-value ${<}0.05$). Overall, our subjects considered BioPortal more useful for supporting their analysis tasks $(\hbox{mean} = 2.13, \hbox{SD} = 1.06)$ than the spreadsheet program $(\hbox{mean} = 3.47, \hbox{SD} = 1.88)$. The observed between-groups difference was statistically significant at the 0.05 level. Thus, our data supported H5.

### F. Effects on Perceived Ease of Use

According to our analysis, the effect of system on perceived ease of use was significant statistically ($p$-value ${<}0.05$). Our subjects considered BioPortal easier to use $(\hbox{mean} = 2.31, \hbox{SD} = 1.06)$ than the spreadsheet program $(\hbox{mean} = 3.24, \hbox{SD} = 0.88)$. The between-groups difference in perceived ease of use was significant at the 0.01 level. Therefore, our data supported H6.

## V. Conclusion

The development of advanced IDI systems and their routine use by public health professionals are becoming increasingly critical. We report here a significant IDI effort, i.e., BioPortal that supports cross-jurisdictional data integration with advanced data query, analysis, and visualization capabilities. We conducted a controlled experiment to evaluate BioPortal along some fundamental system evaluation dimensions and investigated its effects on user task performance, with particular focus on analysis accuracy, task completion efficiency, and user information satisfaction, system, usability, usefulness, and ease of use. Our study generated encouraging findings that suggest desirable effectiveness, usefulness, and ease of use of BioPortal.

We make several contributions to IDI research and practice. First, we designed and implemented an advanced IDI system by addressing essential system development challenges pertinent to data/system integration, analysis support, and visualization. Second, we conducted a controlled experiment to evaluate BioPortal and its impacts on user task performance. Our evaluation had methodological rigor and involved analysis scenarios and tasks common to public health professionals. Third, we are contributing to general practices in public health by providing practitioners with a conveniently accessible, easy-to-use system that enables them to generate better analysis results in less time.

Our future research includes further system enhancements and expanded system evaluations. Both system functionalities and usability need further improvement, including hotspot analysis and such usability dimensions as screen layout and sequence and system information. On the evaluation front, the reported evaluation only considers WNV, botulism, and foot-and-mouth disease and emphasizes frequency- and pattern-related analysis tasks. To better mimic real-world challenges in public health, additional and preferably more diverse analysis scenarios and tasks must be considered in future evaluation studies. While our subject choice is appropriate for the intended evaluation purpose and hypothesis testing, future investigations should also involve public health researchers and practitioners, preferably from different institutions and regions.

## References

[1] Morse S. S., “Factors in the mergence of infectious diseases”, Emerg. Infect. Dis., vol. 1, pp. 7–15, 1995. 8903148 10.3201/eid0101.950102 PMC2626828

[2] Li Y., Yu L. T., Xu P., Lee J. H., Wong T. W., Ooi P. L., and Sleigh A. C., “Predicting super spreading events during the 2003 severe acute respiratory syndrome epidemics in Hong Kong and Singapore”, Amer. J. Epidemiol., vol. 160, pp. 719–728, 2004. 15466494 10.1093/aje/kwh273 PMC7109976

[3] Baker E. L., Potter M. A., Jones D. L., Mercer S. L., Cioffi J. P., Green L. W., Halverson P. K., Lichtveld M. Y., and Fleming D. W., “The public health infrastructure and our nation's health”, Ann. Rev. Pub. Health, vol. 26, pp. 303–318, 2005. 15760291 10.1146/annurev.publhealth.26.021304.144647

[4] Pinner R., Rebmann C., Schuchat A., and Hughes J., “Disease surveillance and the academic, clinical, and public health communities”, Emerg. Infect. Dis., vol. 9, pp. 781–787, 2003. 12890317 10.3201/eid0907.030083 PMC3023420

[5] Chang W., Zeng D., and Chen H., “A novel spatio-temporal data analysis approach based on prospective support vector clustering”, in Proc. Workshop Inform. Technol. Syst., Las Vegas, NV, 2005, pp. 129–134.

[6] Yasnoff W. A., Overhage J. M., Humphreys B. L., and La Venture M. L., “A national agenda for public health informatics”, J. Amer. Med. Inform. Assoc., vol. 8, pp. 535–545, 2001. 11687561 10.1136/jamia.2001.0080535 PMC130064

[7] Kay B. A., Timperi R. J., Morse S. S., Forslund D., McGowan J. J., and Brien T. O, “Innovative information sharing strategies”, Emerg. Infect. Dis., vol. 4, pp. 464–466, 1998. 10.3201/eid0403.980334 PMC2640296 9716975

[8] Weinberg J., “Surveillance and control of infectious diseases at local, national and international levels”, Clin. Microbiol. Infect., vol. 11, pp. 12–14, 2005. 15760438 10.1111/j.1469-0691.2005.01083.x PMC7129597

[9] Sujansky W., “Heterogeneous database integration in biomedicine”, J. Biomed. Inform., vol. 34, pp. 285–298, 2001. 11977810 10.1006/jbin.2001.1024

[10] Zhu B., Ramsey M., and Chen H., “Creating a large-scale content-based airphoto image digital library”, IEEE Trans. Image Process., vol. 9, no. 1, pp. 163–167, Jan. 2000. 18255383 10.1109/83.817609

[11] Kulldorff M., “Prospective time periodic geographical disease surveillance using a scan statistic”, J. Roy. Statis. Soc. A, vol. 164, pp. 61–72, 2001.

[12] Levine N., CrimeStat: A Spatial Statistics Program for the Analysis of Crime Incident Locations, Houston, TX: Ned Levine & Assoc., 2002, and Washington, DC: Nat. Inst. Justice.

[13] Zeng D., Chang W., and Chen H., “A comparative study of spatio-temporal data analysis techniques in security informatics”, in Proc. 7th IEEE Int. Conf. Intell. Trans. Syst., Washington, DC, 2004, pp. 106–111.

[14] Ma J., Zeng D., and Chen H. spatial–temporal cross-correlation analysis: A new measure and a case study in infectious disease informatics Intelligence and Security Informatics, Lecture Notes in Computer Science, Berlin, Germany: Springer, 2006, vol. 3975, pp. 542–547.

[15] DeLone W. H. and McLean E. R., “The DeLone and McLean model of information systems success: A ten-year update”, J. Manage. Inform. Syst., vol. 19, pp. 9–30, 2003.

[16] Rocheleau B., “Evaluating public sector information systems: Satisfaction versus impact”, Eval. Program Plann., vol. 16, pp. 119–129, 1993.

[17] Ives B., Olson M., and Baroudi J. J., “The measurement of user information satisfaction”, Commun. ACM, vol. 26, pp. 785–793, 1983.

[18] Petersen M., Madsen K., and Kjaer A., “The usability of everyday technology: Emerging and fading opportunities”, ACM Trans. Comput.-Human Interact., vol. 9, pp. 74–105, 2002.

[19] Chinga-Alayo E., Huarcaya E., Nasarre C., Aguila R. del, and Llanos-Cuentas A., “The influence of climate on the epidemiology of bartonellosis in Ancash, Peru”, Trans. R. Soc. Trop. Med. Hygiene, vol. 98, pp. 116–124, 2004. 10.1016/s0035-9203(03)00017-8 14964812

[20] Shneiderman B., Designing the User Interface: Strategies for Effective Human–Computer Interaction, 3rd, Reading, MA: Addison-Wesley, 1998.

[21] Davis F. D., “Perceived usefulness, perceived ease of use, and user acceptance of information technology”, MIS Q., vol. 13, pp. 319–339, 1989.

[22] Lee Y., Kozar K., and Larsen K., “The technology acceptance model: Past, present, and future”, Commun. AIS, vol. 12, pp. 752–780, 2003.

[23] Venkatesh V., “Determinants of perceived ease of use: Integrating control, intrinsic motivation, and emotion into the technology acceptance model”, Inform. Syst. Res., vol. 11, pp. 342–365, 2000.

[24] Hu P. J., Zeng D., Chen H., Larson C., Chang W., and Tseng C. Evaluating an infectious disease information sharing and analysis system in Intelligence and Security Informatics, Lecture Notes in Computer Science, Berlin, Germany: Springer, 2005, vol. 3495, pp. 412–417.

[25] Straub D. W., “Validating instruments in MIS research”, MIS Q., vol. 13, pp. 147–169, 1989.

[26] Cohen J., Applied Multiple Regression/Correlation Analysis for the Behavioral Sciences, Hillsdale, NJ: Lawrence Erlbaum Assoc., 1983.

